# Systematic review and meta‐analysis of cervical metastases in oral maxillary squamous cell carcinoma

**DOI:** 10.1002/cnr2.1410

**Published:** 2021-05-08

**Authors:** Clemente Chia, Seraphina Key, Zubair Hasan, Sohaib Virk, Faruque Riffat

**Affiliations:** ^1^ Monash Health Clayton Victoria Australia; ^2^ Monash University Clayton Victoria Australia; ^3^ Westmead Hospital Westmead New South Wales Australia; ^4^ University of Sydney Camperdown New South Wales Australia; ^5^ University of New South Wales Randwick New South Wales Australia; ^6^ Chris O'Brien Lifehouse Camperdown New South Wales Australia; ^7^ Macquarie University Hospital Macquarie University Macquarie Park New South Wales Australia

**Keywords:** alveolar process, carcinoma, hard, lymphatic metastasis, neck dissection, palate, squamous cell

## Abstract

**Background:**

Management of the node‐negative neck in oral maxillary squamous cell carcinoma (SCC), encompassing the hard palate and upper alveolar subsites of the oral cavity, is controversial, with no clear international consensus or recommendation regarding elective neck dissection in the absence of cervical metastases.

**Aim:**

To assess the occult metastatic rate in patients with clinically node negative oral maxillary SCC; both as an overall metastatic rate, and a comparison of patients managed with an elective neck dissection at index surgery, compared to excision of the primary with clinical observation of the neck.

**Methods and results:**

A systematic review was performed by two independent investigators for studies relating to oral maxillary SCC and analysed according to PRISMA criteria. Data were extracted from Pubmed, Ovid MEDLINE, EMBASE, and SCOPUS via relevant MeSH terms. Grey literature was searched through Google Scholar and OpenGrey. Five hundred and fifty‐three articles were identified on the initial search, 483 unique articles underwent screening against eligibility criteria, and 29 studies were identified for final data extraction. Incidence of occult metastases in patients with clinically node negative oral maxillary SCC was identified either on primary elective neck dissection or on routine follow up. Meta‐analyses were performed. Of 553 relevant articles identified on initial search, 29 were included for analysis. The pooled overall rate of occult metastases in patients initially presenting with clinically node‐negative disease was 22.2%. There is a statistically significant effect of END on decreasing regional recurrence demonstrated in this study (RR 0.36, 95% CI 0.24, 0.59).

**Conclusion:**

The results of this systematic review and meta‐analysis suggest elective neck dissection for patients presenting with hard palate or upper alveolar SCC, even in a clinically node negative neck.

## 1 INTRODUCTION

Squamous cell carcinoma (SCC) is the most common malignant tumour within the oral cavity. The primary modality of therapy is surgical excision, with post‐operative radiotherapy for advanced stage tumours or early‐stage tumours with adverse features.^1^ The presence of nodal metastases is thought to be the most significant prognostic factor in head and neck SCC,^2^ and management of the clinically node negative (cN0) neck is guided by the risk of occult metastases or undetectable micro metastases. Based on modelling performed by Weiss et al., recommendations have suggested a 20% risk of cervical metastases as the threshold for treating the N0 neck with elective neck dissection.^3^ Management of the cN0 neck with selective neck dissection of levels I‐III is now well established for most oral cavity subsites, with two large randomised controlled trials in recent years proposing elective neck dissection even in early‐stage oral cavity SCC.^4^, ^5^

The advantages of elective neck dissection include accurate prognostication, and assessment for the requirement for additional therapy such as radiotherapy and chemotherapy.^6^, ^7^ In addition to the prognostic information provided by nodal staging, the presence of extracapsular spread and multiple involved lymph nodes are both adverse prognostic features.^8^

However, management of oral maxillary SCC, encompassing the hard palate and upper alveolar subsites of the oral cavity, presenting with the cN0 neck remains controversial due to a comparative lack in data, and a low metastatic rate in this cohort relative to other oral cavity subsites.^2^ Traditionally, management of the cN0 neck has largely been conservative, with close surveillance.^2^ The maxilla is thought to have limited lymphatic drainage compared to the rich lymphatics of the other subsites of the oral cavity, and oral cavity tumours of the maxilla are thought to be biologically similar to maxillary tumours arising in the sinonasal cavity, where elective neck management is classically not performed.^2^

It has been suggested on recent studies that hard palate SCC behaves as aggressively as other oral cavity subsites, and that the cN0 neck should be managed surgically.^9^, ^10^, ^11^ Given the persisting controversy, this study aims to assess the occult metastatic rate in patients with cN0 oral maxillary SCC; both as an overall metastatic rate, and a comparison of patients managed with an elective neck dissection at index surgery, compared to excision of the primary with clinical observation of the neck.

## 2 MATERIAL AND METHODS

### 2.1 Study identification

An initial search of published literature from database inception to 01 August 2020 was performed using Pubmed, Ovid Medline, Embase, and SCOPUS databases for all English‐language literature. There were no restrictions to age, sex, or ethnicity applied in this search strategy. Grey literature was searched through Google Scholar and OpenGrey. The keywords ‘maxilla’, ‘alveolar process’, ‘hard palate’, ‘squamous cell carcinoma of the head and neck’, ‘head and neck neoplasms’, ‘cervical metastases’, and ‘neck dissection’ or ‘lymph node excision’ were mapped to MeSH terms and searched. A detailed search strategy for each database is available as a supplement (Supplement 1).

**FIGURE 1:**
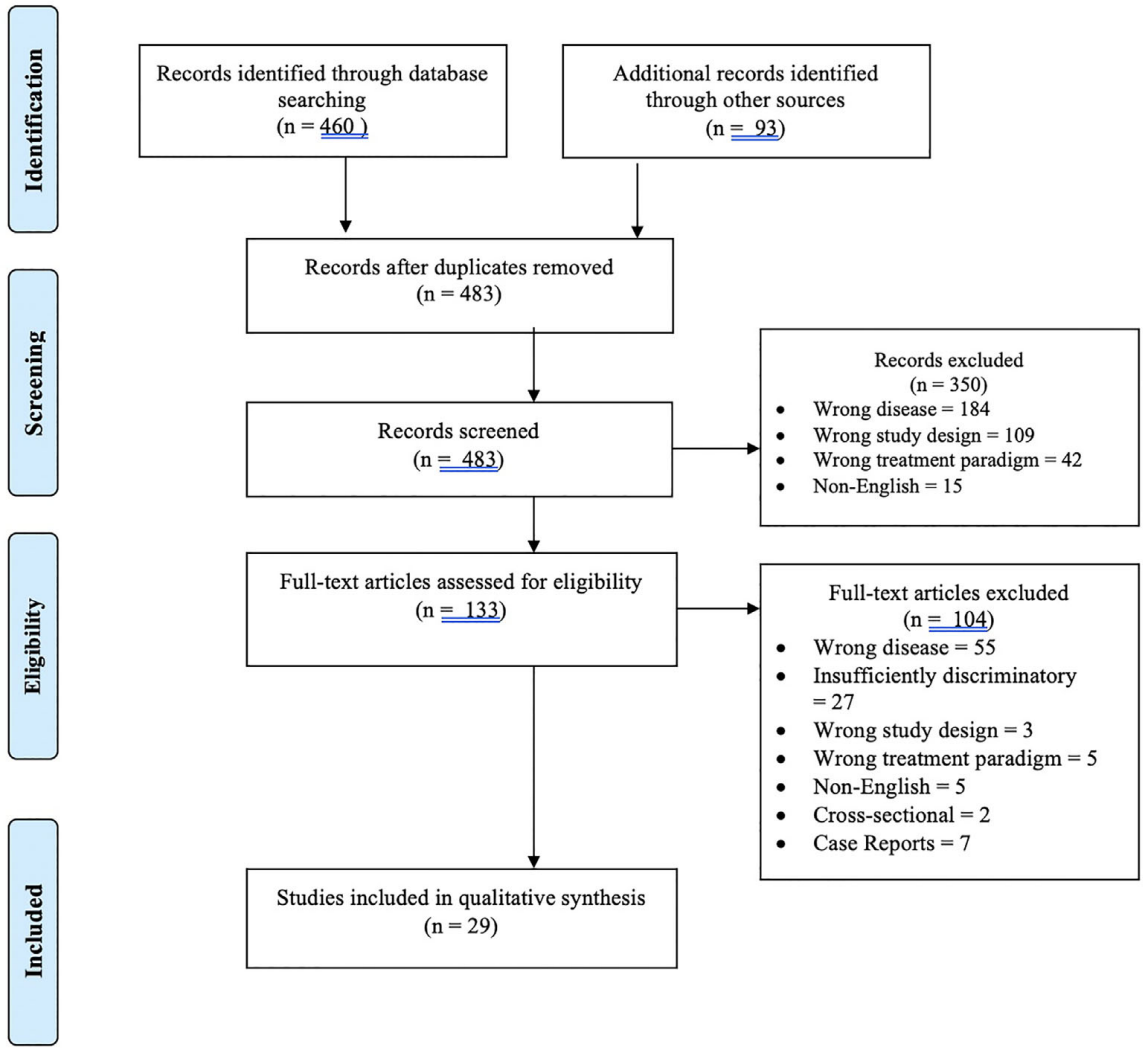
PRISMA flow diagram

The PRISMA flowchart of the study is presented in Figure 1. Two independent reviewers (CC, SK) screened titles and abstracts of the retrieved articles in the initial screening phase. Full articles were obtained for relevant studies, and studies in which the title and abstract provided insufficient information. The individual reference lists of included articles and of similar literature reviews^12^, ^13^, ^14^, ^15^ were also screened for potential inclusion in the next phase.

### 2.2 Study selection

Studies were included according to the following criteria: (1) English language literature, (2) oral cavity tumours of the hard palate, upper alveolar ridge, and maxillary gingiva (3) primary treatment included surgical resection, (4) sample size was greater than five patients.

Studies were excluded based on the following criteria: (1) case reports or small case series with a sample size of five or less, (2) maxillary tumours not originating from the oral cavity subsite of interest, and (3) data was inseparable due to the heterogeneity of patient population. Insufficiently discriminatory data is defined as study data wherein the outcomes of cN0 patients who would otherwise fit inclusion criteria could not be discriminated from clinically node positive (cN+) tumours, tumours invading other sites in the oral cavity, and tumours which could not be discriminated from non‐SCC tumours. All patients who had surgical treatment of their primary were included, regardless of radiotherapy status.

Both reviewers (CC, SK) reviewed the full text of each article, and selected studies for data extraction based on inclusion and exclusion criteria. Both reviewers discussed any differences or discrepancies between final selection, and any further discrepancies were managed in consultation with a third reviewer (ZH).

All studies which fit the inclusion criteria were included into the systematic review. Studies that provided individual patient‐level regional recurrence data stratified into upfront neck dissection and observation subgroups were included into the final meta‐analysis.

### 2.3 Outcomes

The occult cervical metastatic rate of clinically node negative (cN0) patients, determined based on clinical examination and radiological findings, was extracted in three separate subgroups:

1. Occult metastatic rate on pathologically examined nodes following upfront neck dissection (cN0pN+) and;
2. Occult metastatic rate on cN0 patients with upfront neck dissection on long term follow‐up (cN0 and END) and;
3. Occult metastatic rate on cN0 patients without neck dissection on long term follow‐up (cN0 and no END).

Occult metastases were identified in two ways: (1) a neck dissection performed electively at initial surgery for cN0 disease, revealing pN+ disease; and (2) patients with cN0 disease on initial presentation, then presenting with cN+ disease on follow up, after primary surgery. cN0 patients presenting with delayed locoregional recurrence were not included in calculations, as this was thought to be a recurrence of the initial primary, rather than occult nodal metastasis manifesting in delayed cervical lymphadenopathy. The occult metastatic rate at two time points, on initial presentation, and on follow‐up, was calculated.

### 2.4 Secondary outcomes

Predictive and prognostic factors were identified and recorded as secondary outcomes for subgroup analysis: site of primary tumour, early (T1/2) and late (T3/4) stage disease, pathological grade, and survival outcomes.

### 2.5 Data extraction

The following data points were extracted: basic demographics, TNM stage and grade, nodal stage, and follow‐up data. In patients treated with END, pathological nodal status at initial upfront neck dissection was recorded (cN0pN+). As individual studies defined and reported the rates of occult metastasis differently, published data were examined and classified according to this study's definition of occult metastases. For all follow‐up data on our three groups, survival outcomes and nodal recurrence including time to regional recurrence were analysed. Wherein clinical and pathological TNM data was available, clinical tumour staging was used for consistency between studies. Data was pooled to give an overall follow‐up occult metastasis rate across the entire study.

To prevent patient duplication, cN0pN+ patients who proceeded to have regional recurrence were counted as a single event. Data which could not be discriminated between included and excluded patients were excluded from data analysis, such as those where regional recurrence could not be discriminated between patients initially presenting as cN0 and cN+, or wherein hard palate data was mixed with other oral cavity cancer subsites.

Large database studies^16^, ^17^ were excluded due to the heterogeneity of data and inability to extract accurate and sufficient follow up data. Case reports^18^, ^19^, ^20^, ^21^, ^22^, ^23^ were excluded due to insufficient sample size.

The relative risk, odds ratio (OR), risk ratio (RR), or pooled incidence were used as summary statistics and reported with 95% confidence intervals (CI). Meta‐analyses were performed using random‐effected models to take into account the anticipated clinical and methodological diversity between studies. The I^2^ statistic was used to estimate the percentage of total variation across studies due to heterogeneity rather than chance, with values exceeding 50% indicative of considerable heterogeneity.

Publication bias was visually assessed using funnel plots comparing logit of event rates with precision. Egger's linear regression method was used to quantitatively assess for funnel plot asymmetry,^24^ and the Trim‐and‐Fill method was used to explore the impact of studies potentially missing due to publication bias.^25^ Statistical analysis was conducted with Review Manger Version 5.3 (Cochrane Collaboration, Oxford, UK) ad Comprehensive Meta‐analysis v3.0 (Biostat Inc., Englewood, USA). All P‐values were two‐sided, and values <0.05 were considered statistically significant.

### 2.6 Quality appraisal

Risk of bias was assessed by two independent reviewers with the ROBIN‐I tool for the 16 retrospective cohort studies included in meta‐analysis.^26^ Both reviewers discussed any differences or discrepancies, and a graphical representation of the final results was created with the ROBVIS tool.^27^

As all studies included in the meta‐analysis are retrospective observational studies of patient records, general flaws of retrospective cohort studies such as the assumption of accurate recordkeeping apply to studies which did not undergo formal risk of bias assessment. The outcomes of interest, the incidence of nodal disease, and the method of determining regional metastasis on follow‐up, are applicable across most tertiary centres.

## 3 RESULTS

**FIGURE 2:**
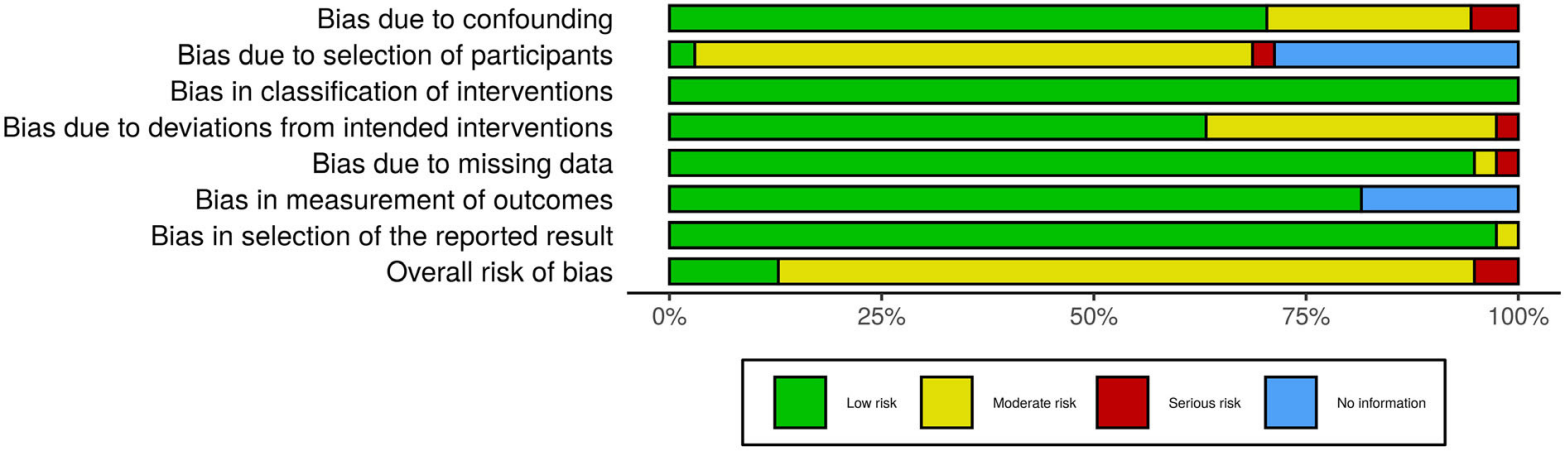
Weighted summary of risk of bias assessment

A total of 553 articles were identified from an initial search: 440 from database searching, five systematic reviews of oral cavity squamous cell cancers with subgroup data specific to upper alveolar subsites; and 38 from hand searching reference lists of these five systematic reviews. Four hundred and eighty‐three unique articles were selected for screening against inclusion and exclusion criteria, and 133 articles met the criteria for full text review. Twenty nine studies underwent data extraction; all were retrospective cohort studies (Oxford Centre for Evidence‐Based Medicine level 3),^28^ and no randomised controlled trials were identified (Figure 1). As all articles selected for full‐text screening and data extraction were available, no authors were contacted. There was moderate risk of bias of 16 retrospective cohort studies included in meta‐analysis (Figure 2), based on a detailed breakdown of individual studies (Supplement 2).

Data pertaining to cN0 patients (n = 1644) from 2001 to 2020 were extracted. These were further analysed in subgroups to examine desired outcomes (Table 1). The sample size and event rate for each data set was recorded (Table 2). Of 1905 patients presenting with maxillary SCC, 86.30% were determined to be clinically node negative. Of all cN0 patients undergoing elective neck dissection, the occult metastatic rate at initial presentation (cN0pN+) is 18.62% (n = 92/494). When these cN0pN+ patients are added to the 261 patients presenting with cN+ disease, the true incidence of nodal disease on initial management is 18.53% (n = 353/1905, 29 studies).

**TABLE 1:** Extractable outcomes for included studies

References	cN0 (n)	Initial presentation		Follow‐up			Subgroup data			
		Incidence of cN0 versus cN+	Initial occult metastatic rate (cN0 with END)	Occult metastatic rate (cN0)	Occult metastatic rate (cN0 with END)^a^	Occult metastatic rate (cN0 with no END)	Primary site	T Stage	Pathological grade	Survival rate
Beltramini et al.^11^	46	1^b^	1	1	1	1	1	1	0^c^	0
Brown et al.^9^	35	1	1	1	1	1	0	1	0	0
Dalal and McLennan^29^	23	1	1	1	1	1	1	1	0	1
Deneuve et al.^30^	52	1	1	1	1	1	0	0	0	0
Eskander et al.^31^	69	1	1	1	1	1	0	1	0	0
Feng et al.^32^	129	0	1	1	1	1	0	1	0	1
Givi et al.^10^	199	0	1	1	1	1	0	0	0	1
Hakim et al.^33^	34	1	1	1	1	1	0	1	0	1
Koshkareva et al.^34^	14	1	1	1	1	1	0	0	0	0
Lubek et al.^35^	35	1	1	0	0	0	0	1	0	0
Montes and Schmidt^36^	11	1	1	1	1	1	1	1	0	1
Montes et al.^37^	124	1	1	1	1	1	0	0	0	0
Moreno‐Sánchez et al.^38^	14	1	0	1	0	1	1	1	1	0
Morris et al.^39^	123	1	1	1	0	0	0	0	0	0
Mourouzis et al.^40^	13	1	1	1	1	1	1	1	0	1
Nicolai et al.^41^	50	1	0	1	0	1	0	0	0	0
Ogura et al.^42^	15	1	0	1	0	1	0	0	0	0
Os et al.^43^	100	1	0	1	0	1	0	0	0	0
Philip et al.^44^	21	1	1	1	1	1	0	1	1	0
Poeschl et al.^45^	74	0	1	1	1	1	0	1	0	1
Qu et al.^46^	107	0	1	1	0	1	1	1	1	1
Salas et al.^47^	54	1	1	1	0	0	0	0	0	0
Simental et al.^48^	23	1	1	1	0	1	0	0	0	0
Valentini et al.^49^	18	1	0	1	0	1	0	0	0	0
Wang et al.^50^	51	1	1	1	1	0	0	0	0	0
Yang et al.^51^	54	1	1	0	0	0	1	1	1	1
Yang et al.^52^	51	1	1	1	0	1	1	1	0	1
Yorozu et al.^53^	14	1	0	1	0	1	0	0	0	0
Zhang et al.^54^	91	1	1	1	1	1	1	0	1	1
Total	1644	1126	494	1555	334	940	410	716	287	786

^a^ Clinically‐node negative (cN0) patients who received upfront elective neck dissection (END), who were found to be pathologically node negative (cN0pN0), and then developed regional recurrence.

^b^ 1: yes.

^c^ 0: no.

**TABLE 2:** Included studies on cervical metastatic rate of oral maxillary squamous cell carcinoma

References	Initial number of patients					Occult metastases on follow up			Total cN0 Occult Met
	Total n	cN0	cN0 + END	cN0 + no END	cN0pN+	cN0 and END	cN0 and no END	All cN0	
Beltramini et al.^11^	54	46	15	31	0	0	6	6	6
Brown^9^	43	35	12	23	3	1	6	7	10
Dalal and McLennan^29^	30	23	6	17	0	0	8	8	8
Deneuve et al.^30^	64	52	3	49	0	0	13	13	13
Eskander et al.^31^	97	69	37	32	11	0	6	6	17
Feng et al.^32^	129	129	50	79	12	2	19	21	33
Givi et al.^10^	199	199	42	157	12	2	30	32	44
Hakim et al.^33^	71	34	22	12	2	0	0	0	2
Koshkareva et al.^34^	20	14	3	11	1	0	0	0	1
Lubek et al.^35^	37	35	35	0	4	NA	NA	NA	4
Montes and Schmidt^36^	14	11	3	8	0	0	2	2	2
Montes et al.^37^	146	124	48	76	10	0	11	11	21
Moreno‐Sánchez et al.^38^	20	14	NA	14	NA	NA	2	2	2
Morris et al.^39^	134	123	8	115	2	NA	NA	22	24
Mourouzis et al.^40^	16	13	1	12	0	0	1	1	1
Nicolai et al.^41^	55	50	NA	50	NA	NA	7	7	7
Ogura et al.^42^	21	15	NA	15	NA	NA	8	8	8
Os et al.^43^	114	100	NA	100	NA	NA	18	18	18
Philip and James^44^	34	21	8	13	0	NA	5	5	5
Poeschl et al.^45^	74	74	36	38	3	6	7	13	16
Qu et al.^46^	107	107	25	78	6	NA	24	24	30
Salas et al.^47^	78	54	3	51	3	NA	NA	14	17
Simental et al.^48^	26	23	3	20	0	NA	7	7	7
Valentini et al.^49^	19	18	NA	18	NA	NA	1	1	1
Wang et al.^50^	55	51	14	37	0	0	10	10	10
Yang et al.^51^	67	54	51	3	5	NA	NA	NA	5
Yang et al.^52^	62	51	35	16	11	NA	3	3	14
Yorozu et al.^53^	19	14	NA	14	NA	NA	3	3	3
Zhang et al.^54^	100	91	34	57	7	4	18	22	29
Total	1905	1644	494	1146	92	17	212	266	358

**FIGURE 3:**
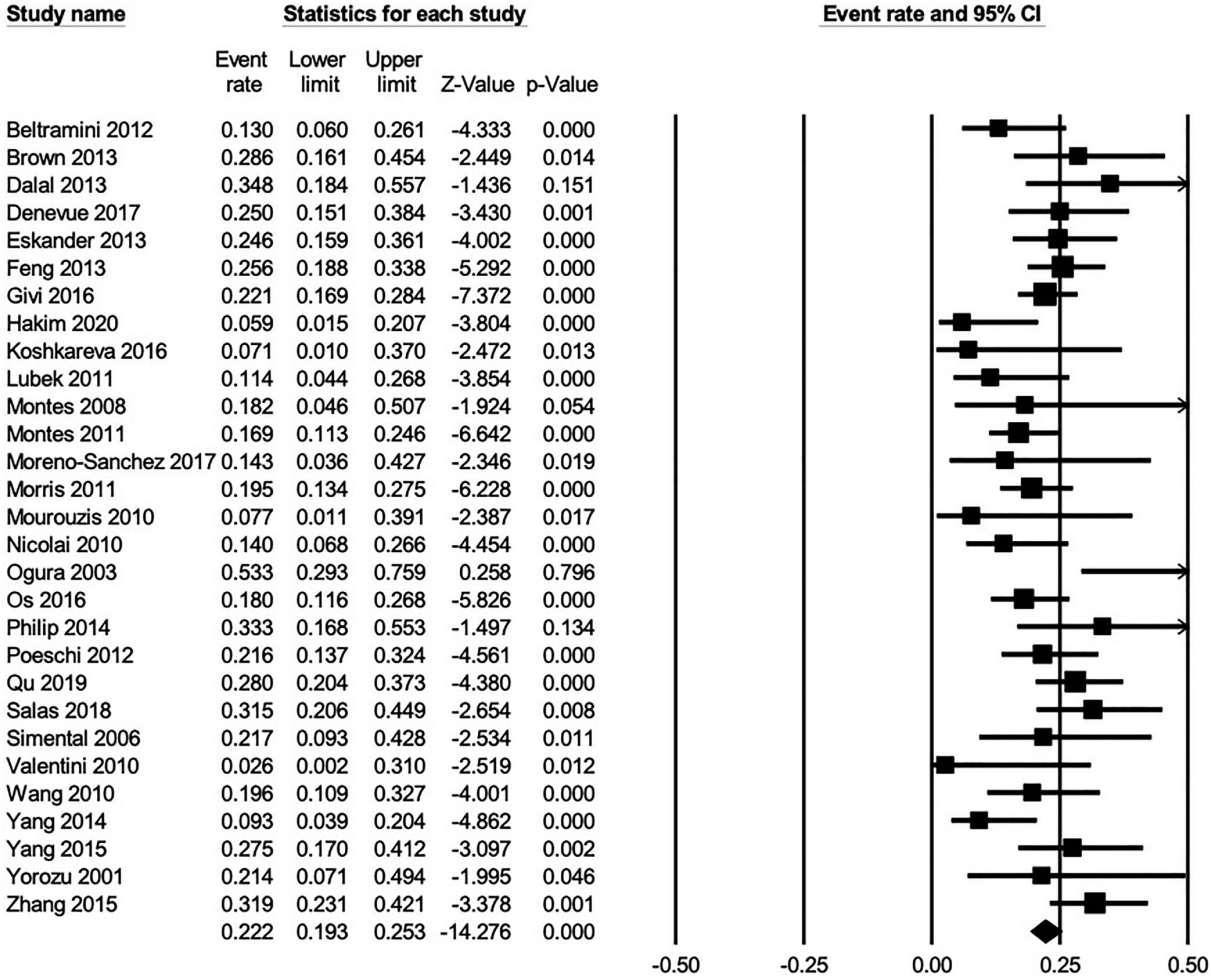
Forest plot for occult metastatic rate in oral maxillary SCC

**FIGURE 4:**
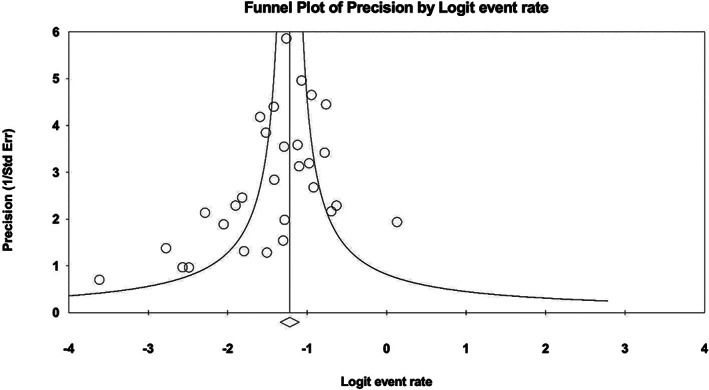
Funnel plot of precision by logit event rate

For patients with upfront elective neck dissection who were found to be pathologically node negative (cN0pN0), the follow‐up recurrence rate was 3.44% (n = 17/494). Hence, the total incidence of nodal disease in patients with upfront END was 22.06% (n = 109/494). For cN0 patients who did not undergo END, the incidence of nodal disease on follow‐up was 18.50% (n = 212/1146). In all patients initially presenting with cN0 disease, the pooled incidence of occult metastases, as a function of cN0pN+ disease identified on index surgery, and patients initially presenting with cN0 disease with nodal disease noted at follow‐up, was 22.2% (95% CI, 19.3‐25.3); I2 = 42%; p for heterogeneity = 0.010 (Figure 3). There was funnel plot asymmetry and a trend towards statistically significant publication bias (*P* = 0.05) using Egger's linear regression method (Figure 4). Using Trim‐and‐Fill method to account for potentially missing studies, the pooled incidence increased to 23.8% (95% CI, 21.8‐26.0).

**FIGURE 5:**
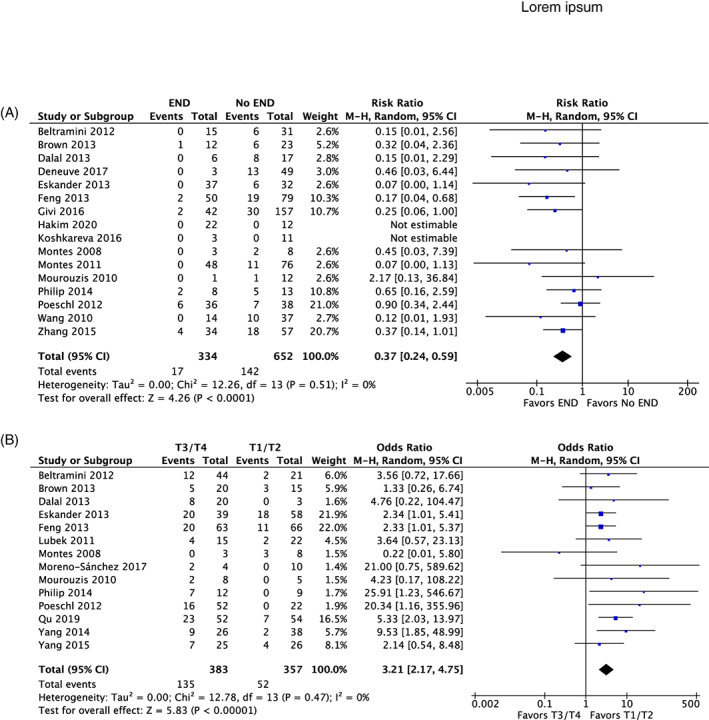
(A) Forest plot for effect of END versus no END on regional recurrence. (B) Forest plot for occult metastatic rate in T1/2 disease versus T3/4 disease

There is a statistically significant effect of END on decreasing regional recurrence (RR 0.36, 95% CI 0.24, 0.59) (Figure 5(A)).

The reported follow‐up period of cN0 patients was pooled to give an average of 50.46 months (SD ±13.94 months, n = 457).^10^, ^32^, ^44^, ^46^ Time to regional recurrence following primary resection (SD ±14.99 months, n = 15, three studies) was pooled to give an average of 9.12 months.^32^, ^36^, ^44^ Average mean follow‐up data for the entire cohort, including both cN0 and cN+ patients, was 49.35 months (SD ±16.47 months, n = 942, 16 studies). Age and gender of each subgroup was unable to be reliably extracted due to heterogenous demographic data.

Secondary outcomes were collected and pooled where available.

Ten studies reported 470 cN0 patients into two subsites: maxillary gingiva, and hard palate. (Table 3) For studies included into this meta‐analysis, nodal disease in the maxillary gingiva was 27.30% (range 0.00%‐46.2%, 10 studies),^11^, ^29^, ^36^, ^38^, ^40^, ^46^, ^51^, ^52^, ^54^, ^55^ and 17.36% (range 0.00%‐33.3%, 8 studies) in the hard palate.^11^, ^29^, ^36^, ^38^, ^40^, ^51^, ^52^, ^54^ An exact number of patients cannot not be given for each subsite, as two papers reported this value in their statistical analysis, and did not give individual patient numbers.^52^, ^54^

**TABLE 3:** Subgroup calculation: Primary tumour site

References	Total	Maxillary gingival			Hard palate		
	n	pN0	pN+	Occult metastasis rate	pN0	pN+	Occult metastasis rate
Beltramini et al.^11^	65	26	19	42.2%	24	5	17.2%
Dalal and McLennan^29^	30	14	7	33.3%	6	3	33.3%
Montes and Schmidt^36^	14	7	6	46.2%	1	0	0.0%
Moreno‐Sánchez et al.^38^	14	4	1	20.0%	8	1	11.1%
Mourouzis et al.^40^	13	6	1	14.3%	5	1	16.7%
Qu et al.^46^	107	77	30	28.0%	0	0	N/A
Yanamoto et al.^55^	1	1	0	0.0%	0	0	N/A
Yang et al.^51^	64	24	7	22.6%	29	4	12.1%
Yang et al.^52^	62	NIL	NIL	32.1%	NIL	NIL	21.7%
Zhang et al.^54^	100	NIL	NIL	34.3%	NIL	NIL	26.7%

Fifteen studies stratified 781 cN0 patients into early‐stage (T1/2) and late‐stage (T3/4) at initial presentation^9^, ^11^, ^29^, ^31^, ^32^, ^33^, ^35^, ^36^, ^38^, ^44^, ^45^, ^46^, ^51^, ^52^ (Table 4). For cN0, occult metastatic rate was 14.17% (range 0.00%‐37.50%, n = 54/381, 15 studies) and 35.25% (range 0.00%‐58.33%, n = 135/381, 14 studies) for early and late T staging respectively, with late‐stage disease having 3.21‐fold increased odds of occult metastases (95% CI 2.17, 4.75) (Figure 5(B)).

**TABLE 4:** Subgroup calculation: T staging

References	Total	T1/2 (of initial cN0)			T3/4 (of initial cN0)		
	n	pN0	pN+	Occult Metastasis Rate	pN0	pN+	Occult Metastasis Rate
Beltramini et al.^11^	65	19	2	9.52%	32	12	27.27%
Brown et al.^9^	35	12	3	20.00%	15	5	35.00%
Dalal and McLennan^29^	23	3	0	0.00%	12	8	40.00%
Eskander et al.^31^	97	40	18	31.03%	19	20	51.28%
Feng et al.^32^	129	55	11	16.67%	43	20	31.75%
Hakim et al.^33^	34	22	2	8.33%	NIL	NIL	NIL
Lubek et al.^35^	37	20	2	9.09%	11	4	26.67%
Montes and Schmidt^36^	8	5	3	37.50%	3	0	0.00%
Moreno‐Sánchez et al.^38^	20	10	0	0.00%	2	2	50.00%
Mourouzis et al.^40^	13	5	0	0.00%	6	2	25.00%
Philip and James^44^	21	9	0	0.00%	5	7	58.33%
Poeschl et al.^45^	74	22	0	0.00%	36	16	30.77%
Qu et al.^46^	107	47	7	12.96%	29	23	44.23%
Yang et al.^51^	67	36	2	5.26%	17	9	34.62%
Yang et al.^52^	51	22	4	15.38%	18	7	28.00%
Total	722	327	54	14.17%	246	137	35.77%

Five studies reported pathological grading for 306 cN0 patients (Table 5).^38^, ^44^, ^46^, ^51^, ^54^ The occult metastatic rate for Grade 1 (well‐differentiated) tumours was 11.18% (range 0.00‐14.71%, n = 19/170, five studies), which was lower than those of Grade 2 and 3 (moderately‐ and poorly‐differentiated), which had an occult metastatic rate of 44.68% (20.0%‐66.67%, n = 63/141, five studies).

**TABLE 5:** Subgroup calculation: Pathological grading for cN0 patients (retrospective cohort)

References	Total	Path grading 1			Path grading 2+		
	n	pN0	pN+	Occult metastasis rate	pN0	pN+	Occult metastasis rate
Moreno‐Sánchez et al.^38^	14	10	0	0.00%	2	2	50.00%
Philip and James^44^	21	11	1	8.33%	3	6	66.67%
Qu et al.^46^	107	54	5	8.47%	28	25	47.17%
Yang et al.^51^	64	29	5	14.71%	24	6	20.00%
Zhang et al.^54^	100	47	8	14.55%	21	24	53.33%

Survival rates were reported where cN0 and cN+ survival rates could be separated (n = 879, 11 studies) (Table 6).^10^, ^29^, ^32^, ^33^, ^36^, ^40^, ^45^, ^46^, ^51^, ^52^, ^54^ The 5‐year overall survival (OS) for cN0 patients (n = 786, 9 studies) was 70.06% (range 45.45%‐91.10%).^10^, ^32^, ^36^, ^40^, ^45^, ^46^, ^51^, ^52^, ^54^ Of studies which reported elective neck dissections as a separate subset, cN0 patients with (n = 136, four studies) and without END (n = 215, five studies) had an average 5‐year OS of 64.21% and 55.89%, respectively.^32^, ^33^, ^36^, ^45^, ^46^ The cN0 5 year disease free survival (DFS) was 45.08% (range 15.00%‐69.57%, n = 235).^10^, ^29^, ^40^ One study (n = 129) reported the DFS for cN0 with and without END as 72.0% and 56.9%, respectively.^32^

**TABLE 6:** Subgroup calculation: Survival rates in cN0 patients

References	cN0		cN0 and END		cN0 and no END	
	5‐year survival	5‐year disease free survival (DFS)	5‐year survival	5‐year DFS	5‐year survival	5‐year DFS
Dalal and McLennan^29^	NIL	69.57%	NIL	NIL	NIL	NIL
Feng et al.^32^	57%	NIL	64.00%	72.00%	52%	56.90%
Givi et al.^10^	68%	50.30%	NIL	NIL	NIL	NIL
Hakim et al.^33^	NIL	NIL	85.70%	NIL	88.90%	NIL
Li et al.^56^	47%	NIL	NIL	NIL	NIL	NIL
Montes and Schmidt^36^	45%	NIL	66.67%	NIL	37.50%	NIL
Mourouzis et al.^40^	69%	15%	NIL	NIL	NIL	NIL
Poeschl et al.^45^	84%	NIL	81%	NIL	56%	NIL
Qu et al.^46^	71%	NIL	(T3/4) 76%	NIL	(T3/4) 46.4%	NIL
Yang et al.^51^	91%	NIL	NIL	NIL	NIL	NIL
Yang et al.^52^	73%	NIL	NIL	NIL	NIL	NIL
Zhang et al.^54^	72%	NIL	NIL	NIL	NIL	NIL

## 4 DISCUSSION

The results of this systematic review and meta‐analysis of 29 studies support the management of hard palate or upper alveolar SCC with elective neck dissection, even in the clinically node negative neck. Within this study, the pooled occult metastatic rate, as a function of both pathological disease on elective neck dissection in cN0 disease and regional recurrence on follow up, was 22.2% This is higher than the accepted occult metastatic rate of 20%,^3^ and supports undertaking a neck dissection at index surgery.

On comparison of subgroups, patients with END at index surgery had 3.44% risk of recurrence on follow up, compared to a recurrence rate of 18.50% in patients with no END at index surgery. Additionally, 5‐year overall survival (OS) rates of cN0 patients with and without END were 64.21% and 55.89%, respectively. This perceived survival benefit further validates END at index surgery.

Late stage (T3/4) tumours had a 35.25% metastatic rate, higher than early stage (T1/2) tumours rate of 14.17%, consistent with the published literature,^13^ with a 3.21‐fold increased odds of occult metastases (95% CI 2.17‐4.75). Additionally, acknowledging a smaller sample size of subgroup analysis, results of this study indicate equivocal nodal recurrence rate in cN0 patients with early stage disease both with and without END. This adds a further consideration in the decision‐making process, suggesting END is indicated for patients presenting with T3‐T4 disease, with no statistically significant results supporting END for T1‐T2 disease.

Patient factors such as age, gender, and comorbidities were unable to be reliably extrapolated from the available studies. It was therefore difficult to analyse the effects of patient factors on survival and selection for END. However, given the inclusion criteria of all patients receiving surgical management of their primary site, it can therefore be postulated that no patient was excluded from END due to being medically unfit for surgery. Additionally, there was a paucity of data relating to specific node levels involved in occult cervical metastases; this was also not able to be reliably extrapolated or analysed from the included sample size.

There was overall moderate risk of bias for the 16 studies included into the final meta‐analysis (Figure 2 *)*. Selection of patients for elective neck dissection appeared inconsistent across the studies analysed, reflecting the lack of a general consensus regarding management of this cohort. Eight studies did not report their criteria in selecting patients for END.^11^, ^31^, ^32^, ^33^, ^34^, ^36^, ^37^, ^44^ Reported indications for neck dissection ranged from surgeon preference,^9^, ^10^, ^29^, ^45^, ^50^ prophylactic clearance as part of a flap reconstruction,^10^, ^30^ and intra‐operative findings.^30^ There was a statistically significant difference in operative technique for one of the studies where free‐flap reconstruction was more likely in the END group.^10^ One study used T4 staging as a factor in choosing to perform END, however only one of six T4 patients had END.^40^ Additionally, due to the role of MDT and individual clinician decisions regarding management following primary resection, patients in the non‐operative arm were either treated with a watch and wait approach, or via radiotherapy in five studies.^10^, ^33^, ^34^, ^36^, ^37^ Additionally, pathological findings of perineural involvement^40^ or positive margins^45^ influenced subsequent management in two studies. For all other studies, there was no further management of the neck. Patient selection for post‐operative radiotherapy to the primary site and subsequent outcomes were not reliably reported across the 29 studies. As such, a separate subgroup analysis on the effects of post‐operative radiotherapy on recurrence and survival outcomes could not be performed.

This study contributes to the existing literature regarding management of a rare subsite in head and neck cancer by providing a detailed and comprehensive systematic review, including meta‐analysis, of 29 retrospective cohort studies. Additionally, this study adds new information to the literature by stratifying patients into those who had treatment of the neck at index surgery and those that did not, and is therefore able to examine the effect of END on nodal recurrence and survival rates. Occult metastatic rates in this study are similar to a previous systematic review performed by Zhang et al., which reported an occult metastatic rate of the cN0 neck of 21% at index surgery, with an overall metastatic rate of 32%, including both cN0pN+ and cN+ patients.^13^ Differences in results between the two systematic reviews can be attributed to differences in study design. Utilising a different study design and tighter exclusion criteria allowed this study to provide more robust data, by including studies not present on previous review, examining the occult metastatic rate at initial diagnosis, and adds new information regarding follow‐up of node‐negative patients and a comparison of nodal recurrence. Several articles from Zhang et al. had to be excluded for various reasons; namely articles that were insufficiently discriminatory between primary subsite and the management of primary and neck, cross‐sectional studies, and the inclusion of oropharyngeal SCC into calculations.^13^ Within this review, studies were not included into subgroup calculations if there was any ambiguity about the exact patient demographic or subgroup, or if it was unclear if the patient had surgical management of the primary. This necessitated exclusion of a number of papers from subgroup analysis which had otherwise robust data.^50^

Two cross‐sectional studies derived from large reporting databases were found on initial literature search pertaining to occult metastatic rates in cN0 patients at initial diagnosis; however, no follow‐up data could be extrapolated from these studies and as such were excluded from meta‐analysis to avoid patient duplication. Lin et al. reported an incidence of cN0 disease at 86.3% from 725 patients, similar to the 86.5% incidence reported in this study. Ninety‐nine patients (13.6%) in this study with either cN0 or cN+ oral maxillary cancer had regional recurrence, however due to the non‐discrimatory nature of the reported data between regional recurrence in node positive and node negative patients, these patients were excluded from meta‐analysis.^17^ Obayemi et al. reported a rate of 14% (n = 59/422) occult metastasis upon neck dissection at primary resection. However, statistical analysis of the study concluded that after controlling for tumour, patient and treatment factors, patients who underwent END had statistically significant improved OS outcomes over an 11‐year period (HR 0.74, *P* = 0.002).^16^

There are several limitations to this review. Many of the studies analysed had were small, retrospective studies with varying levels of heterogeneity in different data points across different studies. Furthermore, data points such as age and gender could not be collected. Although it is hoped this study will influence clinical management of these patients, higher level evidence may come from prospective multi‐institutional data which are currently lacking.

## 5 CONCLUSION

The data in this systematic review and meta‐analysis of 29 articles validate the role of elective neck dissection in hard palate and upper alveolar SCC even in the absence of cervical metastases.

## CONFLICT OF INTEREST

None of the authors have any conflicts of interest or funding sources to disclose.

## AUTHORS' CONTRIBUTIONS

All authors had full access to the data in the study and take responsibility for the integrity of the data and the accuracy of the data analysis. *Conceptualization*, C.C., S.K., Z.H., S.V., F.R.; *Data Curation*, C.C., S.K., S.V.; *Methodology*, C.C., S.K., Z.H., S.V., F.R.; *Investigation*, C.C., S.K., Z.H., S.V.; *Project Administration*, C.C., S.K., Z.H., F.R.; *Formal Analysis*, Z.H., S.V.; *Resources*, F.R.; *Writing ‐ Original Draft*, C.C., S.K.; *Writing ‐ Review & Editing*, C.C., S.K., Z.H., S.V., F.R.; *Visualization*, C.C., S.K.; *Supervision*, Z.H., F.R.; *Software*, S.V.

## ETHICAL STATEMENT

Not applicable.

## Supporting information


Appendix S1: Supplements
Supplement 1: Search strategies
S1.1 MESH Terms
S1.2 Ovid Medline
S1.3 PubMed
S1.4 Scopus
S1.5 Embase
Supplement 2: Risk of Bias Assessment
S2.1 Traffic light plot of authors judgement regarding study quality
S2.2 Individual studies

## Data Availability

The data that support the findings of this study are available from the corresponding author upon reasonable request.
