# Supplementary material for: Systematic review and meta‐analysis of cervical metastases in oral maxillary squamous cell carcinoma
Source: Cancer Rep (Hoboken). 2021 May 8;4(6):e1410. doi: 10.1002/cnr2.1410 (PMC8714539; doi:10.1002/cnr2.1410)
Supplement: Supplementary file 1 — Appendix S1: Supplements Supplement 1: Search strategies S1.1 MESH Terms S1.2 Ovid Medline S1.3 PubMed S1.4 Scopus S1.5 Embase Supplement 2: Risk of Bias Assessment S2.1 Traffic light plot of authors judgement regarding study quality S2.2 Individual studies [file CNR2-4-e1410-s001.docx]

# Supplements

## S1: Search Strategies

**S1.1 MESH Terms**

alveolar process/: "alveolar process"[MeSH Terms] OR ("alveolar"[All Fields] AND "process"[All Fields]) OR "alveolar process"[All Fields]

maxilla/: "maxilla"[MeSH Terms] OR "maxilla"[All Fields] OR "maxillae"[All Fields] OR "maxillas"[All Fields] OR "maxilla's"[All Fields]

palate, hard/: "palate, hard"[MeSH Terms] OR ("palate"[All Fields] AND "hard"[All Fields]) OR "hard palate"[All Fields] OR ("palate"[All Fields] AND "hard"[All Fields]) OR "palate hard"[All Fields]

Gingiva/: "gingiva"[MeSH Terms] OR "gingiva"[All Fields] OR "gingivae"[All Fields]

Squamous Cell: "epithelial cells"[MeSH Terms] OR ("epithelial"[All Fields] AND "cells"[All Fields]) OR "epithelial cells"[All Fields] OR ("squamous"[All Fields] AND "cell"[All Fields]) OR "squamous cell"[All Fields]

Carcinoma, Squamous Cell/: "carcinoma, squamous cell"[MeSH Terms] OR ("carcinoma"[All Fields] AND "squamous"[All Fields] AND "cell"[All Fields]) OR "squamous cell carcinoma"[All Fields] OR ("carcinoma"[All Fields] AND "squamous"[All Fields] AND "cell"[All Fields]) OR "carcinoma, squamous cell"[All Fields]

Lymph Node: "lymph nodes"[MeSH Terms] OR ("lymph"[All Fields] AND "nodes"[All Fields]) OR "lymph nodes"[All Fields] OR ("lymph"[All Fields] AND "node"[All Fields]) OR "lymph node"[All Fields]

Lymph Node Excision/: "lymph node excision"[MeSH Terms] OR ("lymph"[All Fields] AND "node"[All Fields] AND "excision"[All Fields]) OR "lymph node excision"[All Fields]

Lymphatic Metastasis: "lymphatic metastasis"[MeSH Terms] OR ("lymphatic"[All Fields] AND "metastasis"[All Fields]) OR "lymphatic metastasis"[All Fields]

Neck Dissection/: "neck dissection"[MeSH Terms] OR ("neck"[All Fields] AND "dissection"[All Fields]) OR "neck dissection"[All Fields]

Neck: "neck"[MeSH Terms] OR "neck"[All Fields]

sinus: "paranasal sinuses"[MeSH Terms] OR ("paranasal"[All Fields] AND "sinuses"[All Fields]) OR "paranasal sinuses"[All Fields] OR "sinus"[All Fields] OR "sinus's"[All Fields]

**S1.2 Ovid Medline**

1. alveolar process/ or maxilla/ or palate, hard/ or Gingiva/ or Gingiv*.mp.

2. “Squamous Cell Carcinoma of Head and Neck"/ or Squamous Cell Ca*.mp. or Carcinoma, Squamous Cell/

3. head and neck neoplasms/ or facial neoplasms/ or mouth neoplasms/

4. Neck Dissection/ or Neck Dissec*.mp.

5. Lymph Node Excision*.mp. or Lymph Node Excision/ or Lymphatic Metastasis

**S1.3 PubMed**

(((Alveolar Process) OR (maxilla)) OR (Hard palate)) OR (gingiva)

(Squamous Cell Carcinoma) OR (Squamous Cell Carcinoma of Head and Neck)

(((Neck Dissection) OR (Neck Diss*)) OR (Lymph Node Excision)) OR (Lymphatic Metastasis)

Clinical Trial, Meta-Analysis, Randomized Controlled Trial, Review, Systematic Review, Humans, Cancer.

Filters: Humans, Clinical Trials, RCTs, Systematic Review, Meta-Analysis

**S1.4 Scopus**

1. alveolar process/ or maxilla/ or palate, hard/ or Gingiva/ or Gingiv*.mp.

2. “Squamous Cell Carcinoma of Head and Neck"/ or Squamous Cell Ca*.mp. or Carcinoma, Squamous Cell/

3. Neck Dissection/ or Neck Dissec*.mp.

4. Lymph Node Excision*.mp. or Lymph Node Excision/ or Lymphatic Metastasis

5. (AND NOT) sinus*

**S1.5 Embase**

('maxilla'/exp OR 'jaw, upper' OR 'maxilla' OR 'maxillary' OR 'maxillary area' OR 'maxillary growth' OR 'maxillofacial skeleton' OR 'upper jaw' OR 'alveolar ridge'/exp) AND 'head and neck squamous cell carcinoma'/exp AND ('lymph node dissection'/exp OR 'lymph node dissection' OR 'lymph node excision' OR 'lymph node extirpation' OR 'lymph node resection' OR 'lymphadenectomy' OR 'lymphoadenectomy' OR 'neck dissection'/exp) AND ('nodal metastasis'/exp OR 'regional metastasis'/exp OR 'regional metastases' OR 'regional metastasis')

('lymph node dissection'/exp OR 'lymph node dissection' OR 'lymph node dissection, retroperitoneal' OR 'lymph node excision' OR 'lymph node extirpation' OR 'lymph node resection' OR 'lymphadenectomy' OR 'lymphoadenectomy' OR 'retroperitoneal lymph node dissection' OR 'neck dissection'/exp) AND

'nodal metastasis'/exp

4. Lymph Node Excision*.mp. or Lymph Node Excision/ or Lymphatic Metastasis

5. (AND NOT) sinus*

**Supplement 2: Risk of Bias Assessment**

**S2.1 Traffic light plot of authors judgement regarding study quality**


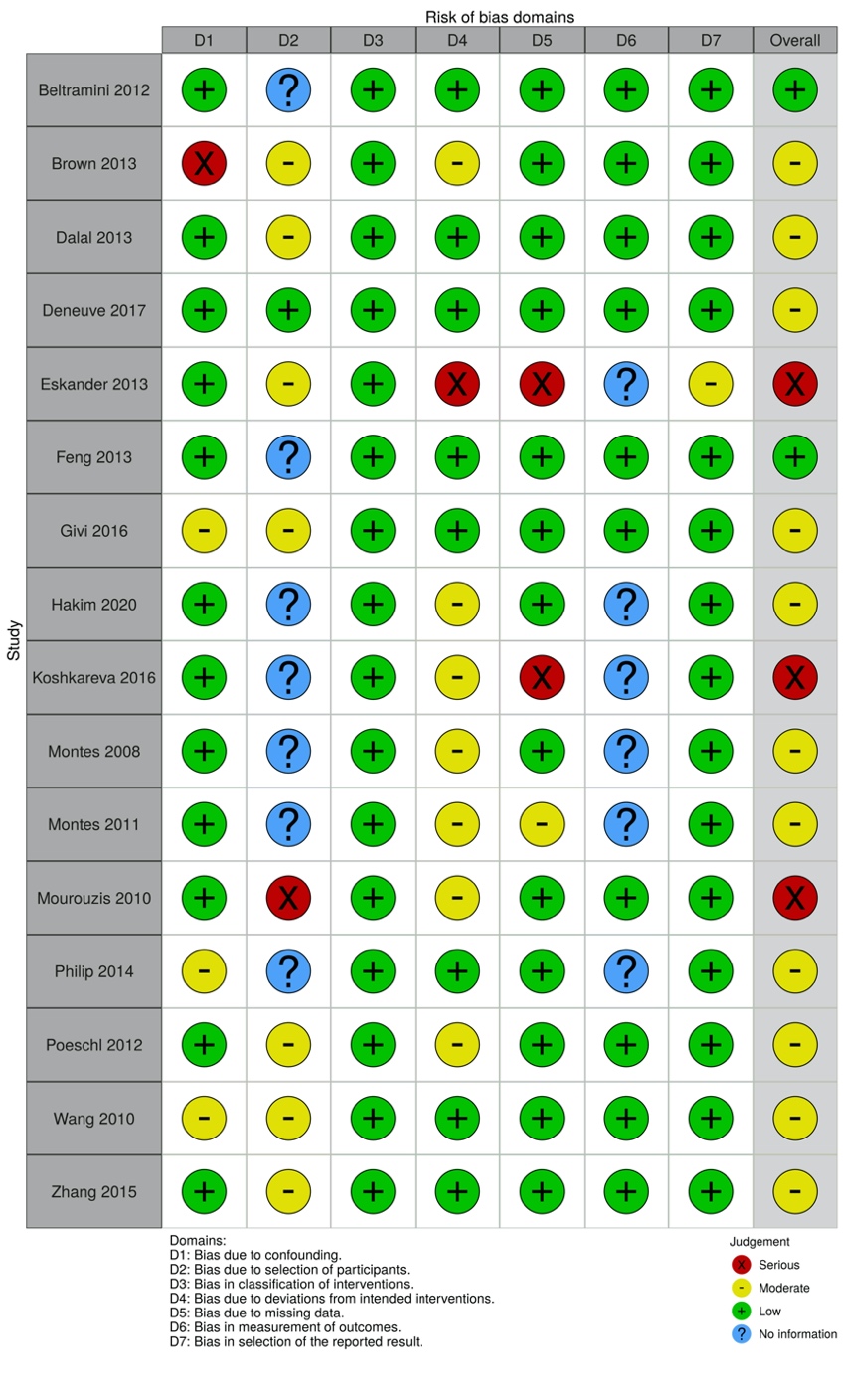


**S2.2 Individual studies**

Beltramini 2012(3)

| Bias | Author’s Judgement | Support for Judgement |
| --- | --- | --- |
| Bias due to confounding | Low | All patients had similar baseline characteristics, surgical management of the primary with no pre-operative chemoradiotherapy. |
| Bias in selection of participants into the study | No information | Selection criteria of patients for elective neck dissection and levels of elective ND not reported. |
| Bias in classification of interventions | Low | cN0 patients tracked in their individual subgroups of END vs no END through oncosurveillance at uniform intervals. |
| Bias due to deviations from intended interventions | Low | All cN0 patients in both upfront ND(cN0pN0) and observation arms received no further management of the neck on follow-up provided no regional recurrence was detected. Regional recurrence not dependent on patient behaviours. |
| Bias due to missing data | Low | Isolated regional recurrence events were reported for patients who had upfront neck dissection and observation. |
| Bias in measurement of the outcome | Low | All regional recurrence detected with clinical review at identical follow-up intervals. |
| Bias in selection of the reported result | Low | Conclusions consistent with the reported data. |

Brown 2013(4)

| Bias | Author’s Judgement | Support for Judgement |
| --- | --- | --- |
| Bias due to confounding | Serious | Patient comorbidities were a pre-operative factor in END vs. no END, and the effect of patient demographics were not accounted for. |
| Bias in selection of participants into the study | Moderate | Selection of patients for elective neck dissection was surgeon-dependent. |
| Bias in classification of interventions | Low | cN0 patients tracked in their individual subgroups of END vs no END through oncosurveillance at uniform intervals. |
| Bias due to deviations from intended interventions | Moderate | Selection of cN0 patients for PORT based on individual MDT assessment. |
| Bias due to missing data | Low | Isolated regional recurrence events were reported for patients who had upfront neck dissection and observation. |
| Bias in measurement of the outcome | Low | All regional recurrence detected with history, FNE, and image-guided biopsy, at identical follow-up intervals. |
| Bias in selection of the reported result | Moderate | Conclusions consistent with the reported data. |

Dalal 2013(5)

| Bias | Author’s Judgement | Support for Judgement |
| --- | --- | --- |
| Bias due to confounding | Low | All patients had similar baseline characteristics, surgical management of the primary with no pre-operative chemoradiotherapy. |
| Bias in selection of participants into the study | Moderate | Selection of patients for elective neck dissection was surgeon-dependent. |
| Bias in classification of interventions | Low | cN0 patients tracked in their individual subgroups of END vs no END through oncosurveillance at uniform intervals. |
| Bias due to deviations from intended interventions | Low | Selection of cN0 patients for PORT based on discussion at MDT as per hospital protocols. |
| Bias due to missing data | Low | Isolated regional recurrence events were reported for patients who had upfront neck dissection and observation. |
| Bias in measurement of the outcome | Low | All regional recurrence detected with history, FNE, and image-guided biopspy, at identical follow-up intervals. |
| Bias in selection of the reported result | Low | Conclusions consistent with the reported data. |

Deneuve 2017(6)

| Bias | Author’s Judgement | Support for Judgement |
| --- | --- | --- |
| Bias due to confounding | Low | All patients had similar baseline characteristics, surgical management of the primary with no pre-operative chemoradiotherapy. |
| Bias in selection of participants into the study | Moderate | Selection of patients for elective neck dissection was surgeon-dependent based on intra-operative findings. |
| Bias in classification of interventions | Low | cN0 patients tracked in their individual subgroups of END vs no END through oncosurveillance at uniform intervals. |
| Bias due to deviations from intended interventions | Low | All cN0 patients in both upfront ND(cN0pN0) and observation arms received no further management of the neck on follow-up provided no regional recurrence was detected. Regional recurrence not dependent on patient behaviours. |
| Bias due to missing data | Low | Isolated regional recurrence events were reported for patients who had upfront neck dissection and observation. |
| Bias in measurement of the outcome | Low | All regional recurrence detected with clinical review at identical follow-up intervals. |
| Bias in selection of the reported result | Low | Conclusions consistent with the reported data. |

Eskander 2013(7)

| Bias | Author’s Judgement | Support for Judgement |
| --- | --- | --- |
| Bias due to confounding | Low | All patients had similar baseline characteristics, surgical management of the primary with no pre-operative chemoradiotherapy. Demographic differences were statistically non-significant. |
| Bias in selection of participants into the study | Moderate | Selection of patients for elective neck dissection was surgeon-dependent. |
| Bias in classification of interventions | Low | cN0 patients tracked in their individual subgroups of END vs no END through oncosurveillance at uniform intervals. |
| Bias due to deviations from intended interventions | Serious | Selection of cN0 patients for PORT based on individual MDT assessment, and unclear which patients received this. |
| Bias due to missing data | Serious | Isolated regional recurrence events not clear for patients with upfront neck dissection. |
| Bias in measurement of the outcome | No information | Unclear how regional recurrence was detected. |
| Bias in selection of the reported result | Moderate | Multiple definitions of occult metastasis were calculated in the paper. |

Feng 2013(8)

| Bias | Author’s Judgement | Support for Judgement |
| --- | --- | --- |
| Bias due to confounding | Low | All patients had similar baseline characteristics, surgical management of the primary with no pre-operative chemoradiotherapy. Demographic differences were statistically non-significant. |
| Bias in selection of participants into the study | No information | Selection criteria of patients for elective neck dissection not reported. |
| Bias in classification of interventions | Low | cN0 patients tracked in their individual subgroups of END vs no END through oncosurveillance at uniform intervals. |
| Bias due to deviations from intended interventions | Low | All cN0 patients in both upfront ND(cN0pN0) and observation arms received no further management of the neck on follow-up provided no regional recurrence was detected. Regional recurrence not dependent on patient behaviours. |
| Bias due to missing data | Low | Isolated regional recurrence events were reported for the two subgroups of interest. |
| Bias in measurement of the outcome | Low | All regional recurrence detected with clinical review at identical follow-up intervals. |
| Bias in selection of the reported result | Low | Conclusions consistent with the reported data. |

Givi 2016(9)

| Bias | Author’s Judgement | Support for Judgement |
| --- | --- | --- |
| Bias due to confounding | Moderate | All patients had similar demographic characteristics and surgical management of the primary. There is a statistically significant difference in pre-operative T staging between the two arms. |
| Bias in selection of participants into the study | Moderate | Selection of patients for elective neck dissection was surgeon-dependent, with statistically significant differences in operative technique including increased free flap reconstruction in the END subgroup. |
| Bias in classification of interventions | Low | cN0 patients tracked in their individual subgroups of END vs no END through oncosurveillance at uniform intervals. |
| Bias due to deviations from intended interventions | Low | Selection of cN0 patients for adjuvant chemoradiotherapy was based on MDT consensus |
| Bias due to missing data | Low | Isolated regional recurrence events were reported for patients who had upfront neck dissection and observation. |
| Bias in measurement of the outcome | No information | Unclear how regional recurrence was detected. |
| Bias in selection of the reported result | Low | Conclusions consistent with the reported data. |

Hakim 2020(10)

| Bias | Author’s Judgement | Support for Judgement |
| --- | --- | --- |
| Bias due to confounding | Low | All patients had similar baseline characteristics, surgical management of the primary with no pre-operative chemoradiotherapy. |
| Bias in selection of participants into the study | No information | Selection criteria of patients for elective neck dissection not reported. |
| Bias in classification of interventions | Low | cN0 patients tracked in their individual subgroups of END vs no END through oncosurveillance at uniform intervals. |
| Bias due to deviations from intended interventions | Moderate | Selection of cN0 patients for adjuvant radiotherapy was based on individual MDT assessment. |
| Bias due to missing data | Low | Isolated regional recurrence events were reported for patients who had upfront neck dissection and observation. |
| Bias in measurement of the outcome | No information | Unclear how regional recurrence was detected. |
| Bias in selection of the reported result | Low | Conclusions consistent with the reported data. |

Koshkareva 2016(11)

| Bias | Author’s Judgement | Support for Judgement |
| --- | --- | --- |
| Bias due to confounding | Low | All patients had similar baseline characteristics, surgical management of the primary with no pre-operative chemoradiotherapy. |
| Bias in selection of participants into the study | No information | Selection criteria of patients for elective neck dissection not reported. |
| Bias in classification of interventions | Low | cN0 patients tracked in their individual subgroups of END vs no END through oncosurveillance at uniform intervals. |
| Bias due to deviations from intended interventions | Moderate | Selection of cN0 patients for adjuvant radiotherapy was based on individual clinician assessment. |
| Bias due to missing data | Serious | Isolated regional recurrence events not clear for patients with upfront neck dissection. |
| Bias in measurement of the outcome | No information | Unclear how regional recurrence was detected. |
| Bias in selection of the reported result | Low | Conclusions consistent with the reported data. |

Montes 2008(12)

| Bias | Author’s Judgement | Support for Judgement |
| --- | --- | --- |
| Bias due to confounding | Low | All patients had similar baseline characteristics, surgical management of the primary with no pre-operative chemoradiotherapy. |
| Bias in selection of participants into the study | No information | Selection criteria of patients for elective neck dissection not reported. |
| Bias in classification of interventions | Low | cN0 patients tracked in their individual subgroups of END vs no END through oncosurveillance at uniform intervals. |
| Bias due to deviations from intended interventions | Moderate | Selection of cN0 patients for adjuvant radiotherapy was based on individual clinician assessment. |
| Bias due to missing data | Low | Isolated regional recurrence events were reported for patients who had upfront neck dissection and observation. |
| Bias in measurement of the outcome | No information | Unclear how regional recurrence was detected. |
| Bias in selection of the reported result | Low | Conclusions consistent with the reported data. |

Montes 2011(13)

| Bias | Author’s Judgement | Support for Judgement |
| --- | --- | --- |
| Bias due to confounding | Low | All patients had similar baseline characteristics, surgical management of the primary with no pre-operative chemoradiotherapy. |
| Bias in selection of participants into the study | No information | Selection criteria of patients for elective neck dissection not reported. |
| Bias in classification of interventions | Low | cN0 patients tracked in their individual subgroups of END vs no END through oncosurveillance at uniform intervals. |
| Bias due to deviations from intended interventions | Moderate | Selection of cN0 patients for adjuvant radiotherapy was based on individual clinician assessment. One surgeon consistently prescribed PORT for all cN0 necks regardless of whether patients received END or no END. |
| Bias due to missing data | Moderate | Isolated regional recurrence events were reported for patients who had upfront neck dissection and observation a/o radiotherapy. |
| Bias in measurement of the outcome | No information | Unclear how regional recurrence was detected. |
| Bias in selection of the reported result | Low | Conclusions consistent with the reported data. A subgroup of patients treated with non-surgical management of the neck via PORT was available. |

Mourouzis 2010(14)

| Bias | Author’s Judgement | Support for Judgement |
| --- | --- | --- |
| Bias due to confounding | Low | All patients had similar baseline characteristics, surgical management of the primary with no pre-operative chemoradiotherapy. |
| Bias in selection of participants into the study | Serious | Selection criteria of patients based on T staging, but inconsistent across study. Of 6 T4 patients, only 1 had END. |
| Bias in classification of interventions | Low | cN0 patients tracked in their individual subgroups of END vs no END through oncosurveillance at uniform intervals. |
| Bias due to deviations from intended interventions | Moderate | Selection of cN0 patients for adjuvant radiotherapy was based on individual clinician assessment for one patient who had perineural involvement. |
| Bias due to missing data | Low | Isolated regional recurrence events were reported for patients who had upfront neck dissection and observation a/o radiotherapy. |
| Bias in measurement of the outcome | Low | All regional recurrence detected with clinical review at identical follow-up intervals. |
| Bias in selection of the reported result | Low | Conclusions consistent with the reported data. |

Philip 2014(15)

| Bias | Author’s Judgement | Support for Judgement |
| --- | --- | --- |
| Bias due to confounding | Moderate | All patients had surgical management of the primary with no pre-operative chemoradiotherapy, however the T staging was higher(T4) for the one patient with END |
| Bias in selection of participants into the study | No information | Selection criteria of patients for elective neck dissection not reported. |
| Bias in classification of interventions | Low | cN0 patients tracked in their individual subgroups of END vs no END through oncosurveillance at uniform intervals. |
| Bias due to deviations from intended interventions | Low | All cN0 patients in both upfront ND(cN0pN0) and observation arms received no further management of the neck on follow-up provided no regional recurrence was detected.. Regional recurrence not dependent on patient behaviours. |
| Bias due to missing data | Low | Isolated regional recurrence events were reported for patients who had upfront neck dissection and observation. |
| Bias in measurement of the outcome | No information | Unclear how regional recurrence was detected. |
| Bias in selection of the reported result | Low | Conclusions consistent with the reported data. |

Poeschl 2012(16)

| Bias | Author’s Judgement | Support for Judgement |
| --- | --- | --- |
| Bias due to confounding | Low | All patients had similar baseline characteristics, surgical management of the primary with no pre-operative chemoradiotherapy. |
| Bias in selection of participants into the study | Moderate | Selection of patients for elective neck dissection was surgeon-dependent, with surgeons preferentially performing neck dissection for T4 tumours |
| Bias in classification of interventions | Low | cN0 patients tracked in their individual subgroups of END vs no END through oncosurveillance at uniform intervals. |
| Bias due to deviations from intended interventions | Moderate | 4 patients with positve margins had re-do surgeries. All cN0 patients in both upfront ND(cN0pN0) and observation arms received no further management of the neck on follow-up, hence no difference in clinician behaviour. Regional recurrence not dependent on patient behaviours. |
| Bias due to missing data | Low | Isolated regional recurrence events were reported for patients who had upfront neck dissection and observation. |
| Bias in measurement of the outcome | Low | All regional recurrence detected with clinical review at identical follow-up intervals. |
| Bias in selection of the reported result | Low | Conclusions consistent with the reported data. |

Wang 2010(17)

| Bias | Author’s Judgement | Support for Judgement |
| --- | --- | --- |
| Bias due to confounding | Moderate | All patients had similar baseline characteristics and surgical management of the primary. There is a difference in pre-operative stage between the two arms, wherein patients with upfront END were more likely to have Stage III and IV disease. Patients where disease involved the posterior hard palate were included into the study, and could not be removed from the analysis. |
| Bias in selection of participants into the study | Moderate | Selection of patients for elective neck dissection was surgeon-dependent. |
| Bias in classification of interventions | Low | cN0 patients tracked in their individual subgroups of END vs no END through oncosurveillance at uniform intervals. |
| Bias due to deviations from intended interventions | Low | All cN0 patients in both upfront ND(cN0pN0) and observation arms received no further management of the neck on follow-up provided no regional recurrence was detected.. Regional recurrence not dependent on patient behaviours. |
| Bias due to missing data | Low | Isolated regional recurrence events were reported for patients who had upfront neck dissection and observation. |
| Bias in measurement of the outcome | Low | All regional recurrence detected with history, FNE, relevant imaging, and image-guided biopspy, at identical follow-up intervals. |
| Bias in selection of the reported result | Low | Conclusions consistent with the reported data. |

Zhang 2015(18)

| Bias | Author’s Judgement | Support for Judgement |
| --- | --- | --- |
| Bias due to confounding | Low | All patients had similar baseline characteristics, surgical management of the primary with no pre-operative chemoradiotherapy. |
| Bias in selection of participants into the study | Moderate | Selection criteria of patients for elective neck dissection became more common as with chronological time. |
| Bias in classification of interventions | Low | cN0 patients tracked in their individual subgroups of END vs no END through oncosurveillance at uniform intervals. |
| Bias due to deviations from intended interventions | Low | All cN0 patients in both upfront ND(cN0pN0) and observation arms received no further management of the neck on follow-up provided no regional recurrence was detected. Regional recurrence not dependent on patient behaviours. |
| Bias due to missing data | Low | Isolated regional recurrence events were reported for patients who had upfront neck dissection and observation. |
| Bias in measurement of the outcome | Low | All regional recurrence detected with history, relevant imaging, and biopspy, at identical follow-up intervals. |
| Bias in selection of the reported result | Low | Conclusions consistent with the reported data. |
